# Lead and Cadmium Content in Grass Growing Near An Expressway

**DOI:** 10.1007/s00244-018-0565-3

**Published:** 2018-09-26

**Authors:** Kazimierz Jankowski, Elżbieta Malinowska, Grażyna A. Ciepiela, Jolanta Jankowska, Beata Wiśniewska-Kadżajan, Jacek Sosnowski

**Affiliations:** Faculty of Natural Science, Siedlce University of Natural Sciences and Humanities, Siedlce, Poland

## Abstract

The purpose of the study was to evaluate the effect of distance from a road on lead and cadmium content in grass species near an expressway and to assess bioaccumulation of these elements by morphological parts of the plants. The material for the research was the following grass species in their flowering stage: *Dactylis glomerata, Arrenatherum elatius*, and *Alopecurus pratensis.* Plant samples were collected along the international E30 road, the ring-road of Siedlce, in May 2015. A 9-km road section was examined with samples collected on both sides, covering a stretch of 700 m, at the following distances from the edge of the road: 1, 5, 10, and 15 m. Five samples of each plant species and at each distance from the road were collected. Lead and cadmium concentration was determined with the AAS method. The largest amounts of Pb were absorbed by *A*. *pratensis* L. (3.843 mg kg^−1^DM), while the lowest by *A*. *elatius* L. (2.523 mg kg^−1^DM). Of the above plants, the highest amount of Cd (0.286 mg kg^−1^DM) was accumulated by *D*. *glomerata* L. Underground parts of the grass species accumulated greater amounts of Pb and Cd than aboveground parts. It indicates that considerable amounts of heavy metals released by expressway vehicles contaminated the soil. The highest content of Pb and Cd was found in the grass growing at a distance of 5 m from the edge of the roadway, and this applies both to underground and aboveground parts.

Heavy metal contamination is a major concern, because it can lead, for example, to their bioaccumulation in the food chain, which affects human health (Peralta-Videa et al. 2009), to an inhibition of biodegradation of organic contaminants (Maslin and Maier 2000; Alloway 2013), to groundwater contamination (Mulligan et al. 2001), and to reduction of land and food quality (McLaughlin et al. 2000; Kabata-Pendias 2004; Gardea-Torresdey et al. 2005; Martin et al. 2017). According to Cicek et al. (2012), pollution from roadways and automobiles is now considered to be one of the largest sources of heavy metals. The contribution of road transport to the global emission of atmospheric pollutants is regularly increasing (Vachova et al. 2017; Cicek et al. 2012; Serbula et al. 2012).

The most important sources of heavy metals are brake lining wear, exhaust fumes, tire wear, crash barrier corrosion (Adachia and Tainosho 2004; McKenzie et al. 2009), or motor oil (Olajire and Ayodele 1997). Heavy metals, including lead and cadmium with the strongest impact on the environment, are among many chemical substances deposited near roads. However, their actual accumulation in plants is mainly connected with the bioaccumulation potential, which is species-specific (Jankowski et al. 2014).

Literature review in this field reveals that a variety of plants are used in monitoring accumulations of heavy metals, and most of them have produced successful results (Tomasevic et al. 2004; Chakrabortty and Paratkar 2006; Vachova et al. 2017). According to Cicek et al. (2012), more than 400 plant species, known as hyperaccumulators, from all over the world can accumulate high concentrations of metals at contaminated sites. The diverse ability of plants to accumulate heavy metals in aboveground parts is due to different morphology of plants (Deska et al. 2011; Jankowski et al. 2014). The structures on leaf surfaces, grooves, bristles, or specific chemicals, such as wax, play a major role in this process (Naszradi et al. 2004). They accumulate dust on the surface of plants in a variety of ways. There also are differences in accumulation of heavy metals in different parts of plants (Stafilov and Jordanovska 1997).

Some authors (Jankowski et al. 2014; Malinowska et al. 2015; Wiśniewska-Kadżajan et al. 2015) point out that of all plants grasses are typical groundcovers in areas adjacent to a roadway. Roadside sward is composed of grasses, which are usually planted there, but also of dicotyledonous herbs and weeds. The fact that grass can grow very quickly, even in small areas, and that it is an all-season plant is a reason why it was used in some studies in a very successful way to monitor pollution (Olajire and Ayodele 1997; Garcia and Milan 1998; Lai and Chen 2004; Filipek-Mazur et al. 2007; Cicek et al. 2012). Emission of heavy metals (lead and cadmium) coming from roads can cause diverse pollution of grass species and their morphological parts (De Nicola et al. 2003; Naszradi et al. 2004; Jankowski et al. 2015).

Heavy metals released by traffic pose the greatest threat to areas directly adjacent to roads. Road dust is a mixture of pollutants floating through the air, but it is also washed off road surfaces by rainwater or splashed by the wheels of moving cars onto the roadside (Werkenthin et al. 2014). The dust contains significant amounts of polycyclic aromatic hydrocarbons (2.7 Mg per year, which is 1–1.9% of total emission). In the EU countries, the annual air emission of lead (Pb) is estimated at 2.4 Gg and cadmium (Cd) at 105 Mg. Poland’s share in this emission is very significant, with 23% for Pb and 41% for Cd (European Environment Agency 2011). Road transport in the EU is responsible for 6% of the total emissions of Pb into the air (Pulles et al. 2012). The problem of environmental pollution associated with road transport continues to be complex, and it has not been studied exhaustively yet.

The purpose of the study was to evaluate the effect of distance from a fast road on the content of lead and cadmium in grass species and to assess bioaccumulation of these elements by morphological parts of the grasses.

## Materials and Methods

**Fig. 1:**
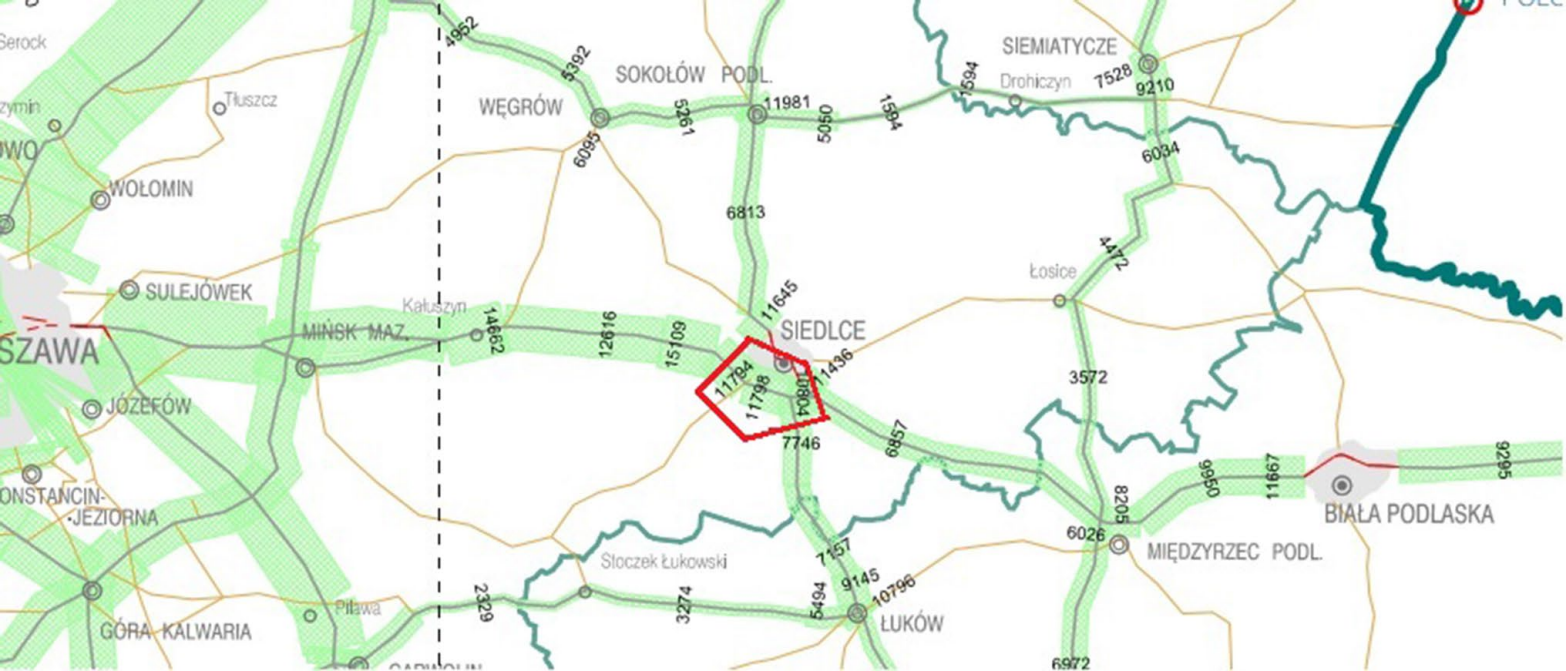
Schematic representation of the studied sites of the E30 road (General Directorate 2015)

**Fig. 2:**
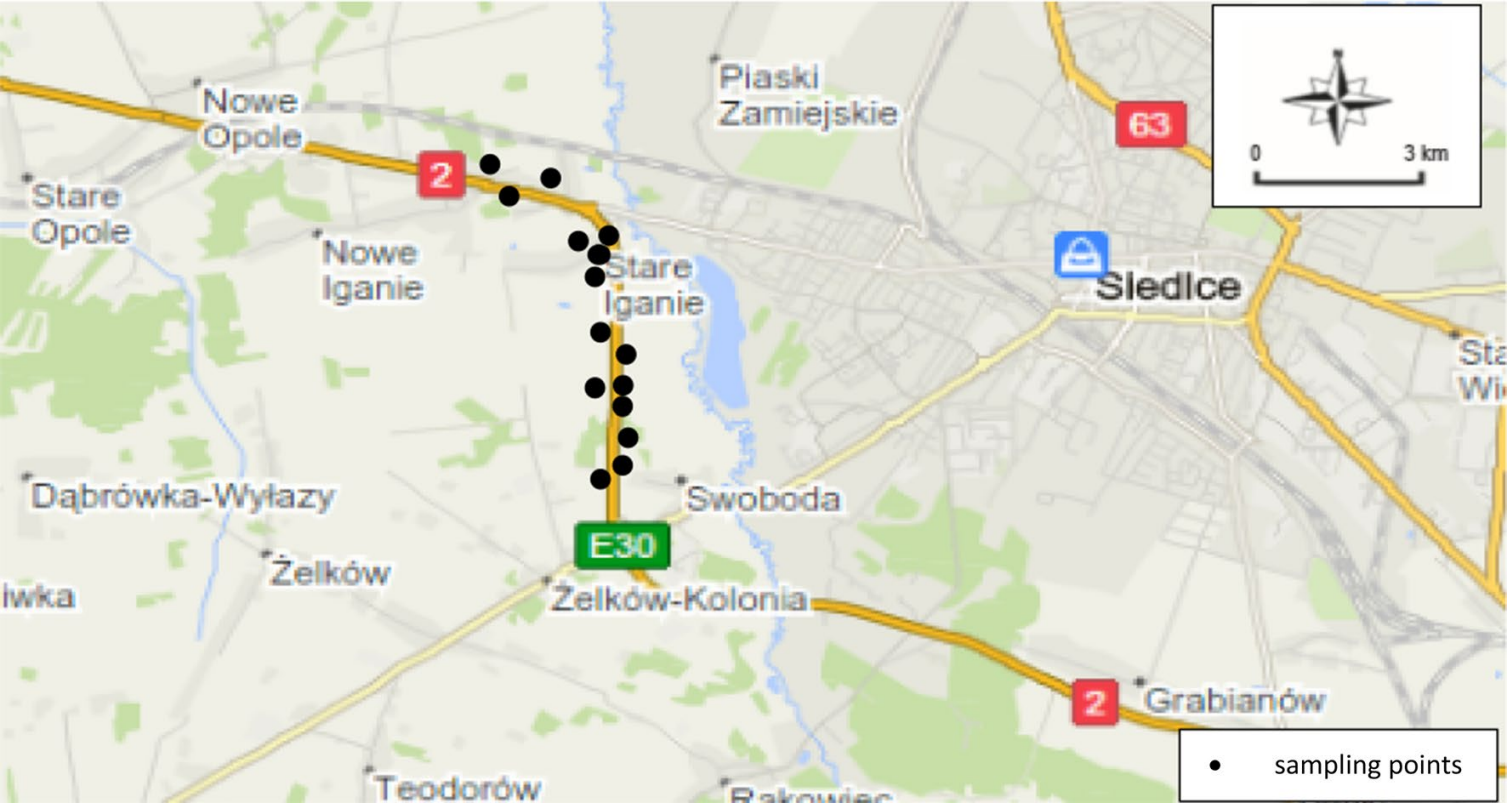
Plant and soil sampling sites in the vicinity of the E30 expressway (openstreetmap.org/#map = 12/52.1036/22.1357)

The plant material for the research was morphological parts of the following grass species in their flowering stage: *Dactylis glomerata, Arrenatherum elatius,* and *Alopecurus pratensis.* Samples of plants were collected along the international E30 road, the ring-road of Siedlce (Figs. 1, 2) in May 2015. A 9-km road section was examined with samples collected on both sides covering a stretch of 700 m at the following distances from the edge of the road: 1, 5, 10, and 15 m. The total number of samples was 96.

The E30 road is a part of the international traffic route from Cork (Ireland) to Omsk (Russia). In Poland, it passes through five provinces, but the range of the research covered only a section of the road in the Masovian province. The road section is located in the central-eastern part of Poland, approximately 80 km east of Warsaw. In 2015, the General Directorate for National Roads and Motorways (GDNR & M) counted the number of vehicles (SDR) on Polish roads, including the tested section (Table 1). The average of three counts of the daily number of motor vehicles (SDR) on international roads in 2015 was 20,067 vehicles/day, whereas on remaining national roads it was 7614 vehicles/day (General Directorate 2015). On the analysed section, the ring-road of Siedlce, the average daily movement of motor vehicles was higher than the average on national roads of Poland, and it was 11,465 vehicles/day (General Directorate 2015).

**Table 1:** The average daily traffic of motor vehicles within the ring road of Siedlce (General Directorate 2015)

Measuring point number	Distance between measuring points (km)	Measuring point name	The number of vehicles per 24 h	Motorcycles	Passenger cars	Vans	Lorries		Coaches
							Without trailers	With trailers	
11,517	5.59	Siedlce/Ring road 1	11,794	63	8420	936	534	1793	47
11,518	5.04	Siedlce/Ring road 2	11,798	36	7718	1240	628	2139	36
11,504	2.55	Siedlce/Ring road 3	10,804	34	7264	1017	540	1912	36
	Means		11,465	44	7800	1064	567	1948	40

The average concentration of organic carbon in the soil sampled 1, 5, 10, and 15 m from the road was 9.05 g kg^−1^, while total nitrogen concentration was 0.57 g kg^−1^, and pH_KCl_ was 4.10. The abovementioned samples were divided according to two morphological parts: aboveground and underground. Then, five samples of each plant species and from each distance from the road were selected. Next, plant material was dried in an oven (SUSLAB–PLE-406, SLW 1000 STD) at 105 °C for 48 h. It was ground to 0.25 mm particle size with 1 g samples weighed out and poured into stoneware crucibles, and then organic matter was ashed at 450 °C in a muffle furnace (L3/11) for 18 h, diluted in 10% HNO_3_, and transferred to a 100-ml volumetric flask.

Lead and cadmium concentration was determined with the AAS method, by means of the Varian Spectra AA20 spectrophotometer (VARIAN, Australia), using the standards of the Merck company. The parameters of the spectrophotometer are: VARIAN, Australia; range of 190 nm to 900 nm, equipped with a graphite cuvette type GTA-96.

An internal quality control system was used for validation of analytical methods. It was assumed that the average recovery of an internal standard should be in the range of 85% to 115% of the true value. For the needs of the research, two standards of quality control were prepared. One standard was added to a known amount of analyte. A recovery was calculated after the analysis of the sample with and without the analyte. The average recovery was dependent on the analyte content in the sample. The analysis was performed in each series of samples, and the obtained recovery was included in the assumed range.

To assess the impact of road traffic on the environment, the pollution index of heavy metals in the soil (*P*_*i*_) was determined (European Environment Agency 2011). It was calculated according to the formula: *P*_*i*_ =  $\frac{{C_{P} }}{{C_{u} }}$

[*C*_*p*_—the concentration of a metal in a polluted sample; *C*_*u*_—the concentration of a metal in a sample representing an unpolluted area with similar characteristics, for Cd it is 0.13 mg kg^−1^, and for Pb it is 7.10 mg kg^−1^ (Czarnowska 1996)].

*P*_*i*_ values are classified in the three-range scale:

- *P*_*i*_ ≤ 1 low level pollution,
- 1 ≤ *P*_*i*_ ≤ 3 medium-level pollution,
- *P*_*i*_ > 3 high level pollution.

Simple correlation coefficient between the overall content of lead and cadmium in the soil and the content of these elements in above-ground and underground parts of selected grass species was also determined.

## Statistical Analysis

The obtained data were processed statistically with the Statistica software, Version 10.0 StatSoft. The effects of the tested factors on lead and cadmium pollution was examined with two-factor analysis of variance. For the purpose of detailed comparison of means, Tukey’s test was carried out at *p* ≤ 0.05.

## Results

The average content of Pb and Cd in the soil sampled at all distances from the expressway was 10.37 and 0.221 mg kg^−1^, respectively (Table 2). In the present study, the highest amounts of Pb (13.52 mg kg^−1^) and Cd (0.333 mg kg^−1^) were found in the soil at a sampling distance of 5 m from the roadway, but such concentration was not toxic to plants.

**Table 2:** Average concentration of Pb and Cd in the soil at all sampling distances from the expressway

Distance from the roadway (m)	Average concentration in the soil (mg kg^−1^)	
	Pb	Cd
1	7.28 D	0.189C
5	13.52 A	0.333A
10	10.65 B	0.210B
15	10.04 C	0.152D
Means	10.37	0.221

Within a column, different uppercase letters indicate a significant difference

The content of both Pb and Cd in the aboveground parts of plants (Table 3) significantly varied and was dependent on both the distance from the roadway and grass species. Pb concentration ranged from 1.049 mg kg^−1^DM in *Arrhenatherum elatius* collected at a distance of 15 m from the roadway to 4.730 mg kg^−1^DM in *D*. *glomerata* collected 5 m from the roadway. Of all the species, the greatest amount of Pb (3.843 mg kg^−1^DM) was absorbed by *A*. *pratensis* and the smallest (3.246 mg kg^−1^DM) by *D*. *glomerata*. Differences in Pb content between grass species were statistically significant.

**Table 3:** Concentration of Cd and Pb in the aboveground parts of selected grass species

Distance from the road (m)	Grass species	Concentration (mg kg^−1^)	
		Pb	Cd
1	*D. glomerata*	2.945	0.237
	*A. elatius*	2.050	0.156
	*A. pratensis*	2.859	0.095
5	*D. glomerata*	4.730	0.537
	*A. elatius*	3.895	0.204
	*A. pratensis*	3.489	0.187
10	*D. glomerata*	2.974	0.227
	*A. elatius*	3.097	0.329
	*A. pratensis*	4.517	0.144
15	*D. glomerata*	2.335	0.141
	*A. elatius*	1.049	0.069
	*A. pratensis*	4.506	0.139
Means for each distance			
1		2.618 C	0.163 C
5		4.038 A	0.309 A
10		3.529 B	0.233 B
15		2.630 C	0.116 D
Means for each species			
*D. glomerata*		3.246 B	0.286 A
*A*. *elatius*		2.523 C	0.190 B
*A. pratensis*		3.843 A	0.141 C

Within a column, different uppercase letters indicate a significant difference

Of all samples collected at different distances from the road the smallest amount of Pb, which was 2.618 mg kg^−1^DM, was found in plants growing 1 m from the road, and the greatest amount of 4.038 mg kg^−1^DM was found at the next sampling point, 5 m from the road. Further away from the expressway Pb content in plants underwent systematic reduction. Differences between Pb content in aboveground parts sampled at different distances were statistically significant. Of all grass species and all distances from the expressway, it was found that the aboveground parts of *D*. *glomerata* and *A*. *elatius* accumulated most Pb when growing 5 m from the roadway, and the quantity of this chemical element decreased as their distance from the road increased. In turn, in *A*. *pratensis* Pb content increased when the distance from the road increased, reaching its highest concentration of 4.517 mg kg^−1^DM at the third distance, namely 10 m from the road.

In the present study, Cd content in aboveground parts of grass (Table 3) was much lower than in the case of Pb. The amount of Cd significantly varied and was dependent on both the grass species and distance from the road. The concentration of this metal ranged from 0.069 mg kg^−1^DM in *A*. *elatius* sampled at a distance of 15 m from the road to 0.537 mg kg^−1^DM in *D*. *glomerata* collected 5 m from the road.

Comparing Cd concentration in all species, it was found that its greatest quantities of 0.286 mg kg^−1^DM were accumulated by *D*. *glomerata*, and the smallest, namely 0.141 mg kg^−1^DM, by *A*. *pratensis*. Differences between Cd content in the aboveground parts were statistically significant. As in the case of Pb, the largest quantities of Cd were accumulated by plants growing at a distance of 5 m from the road (0.309 mg kg^−1^ DM), whereas at further distances (10 and 15 m) the quantities systematically decreased.

As regards the interaction between grass species and sampling distances from the road, it was found that *D*. *glomerata* and *A*. *pratensis* accumulated the highest amounts of Cd when they grew 5 m from the road (0.537 and 0.187 mg kg^−1^DM, respectively), whereas *A*. *elatius* had the highest Cd concentration at a further distance, 10 m from the road (0.329 mg kg^−1^DM).

To assess plant quality to be used as forage for animals, determination of the content of heavy metals was made in accordance with the Regulation of the Minister of Agriculture and Rural Development of 23 January 2007 on the permissible content of undesirable substances in fodder (Regulations of the Minister for Agriculture and Rural Development 2007).

By comparing Pb and Cd content in the underground and aboveground parts of grass species, it was found that the amount of these chemical elements was almost twice higher in the former than in the latter. However, the content of Pb and Cd in the underground parts was significantly differentiated, depending on the grass species and distance from the road (Table 4). The species had similar abilities to accumulate Cd and Pb both in the roots and in the aboveground parts. On average, the highest content of Pb was in the roots of *A*. *elatius* (6.233 mg kg^−1^), and the highest content of Cd was in the underground parts of *D*. *glomerata* (0.538 mg kg^−1^). The highest content of both heavy metals in underground parts was found in grass collected at a distance of 5 m from the road.

**Table 4:** Concentration of Cd and Pb in the underground parts of selected grass species

Distance from the road (m)	Grass species	Concentration mg kg^−1^	
		Pb	Cd
1	*D. glomerata*	4.975	0.489
	*A. elatius*	3.541	0.379
	*A. pratensis*	5.249	0.426
5	*D. glomerata*	6.760	0.789
	*A. elatius*	6.125	0.427
	*A. pratensis*	5.879	0.516
10	*D. glomerata*	5.004	0.479
	*A. elatius*	5.327	0.552
	*A. pratensis*	6.907	0.473
15	*D. glomerata*	4.365	0.393
	*A. elatius*	3.279	0.292
	*A. pratensis*	6.896	0.468
Means for each distance			
1		4.588 D	0.434 C
5		6.255 A	0.577 A
10		5.746 B	0.501 B
15		4.847 C	0.384 D
Means for each species			
*D. glomerata*		5.276 B	0.538 A
*A. elatius*		4.568 C	0.413 C
*A. pratensis*		6.233 A	0.471 B

Within a column, different uppercase letters indicate a significant difference

**Fig. 3:**
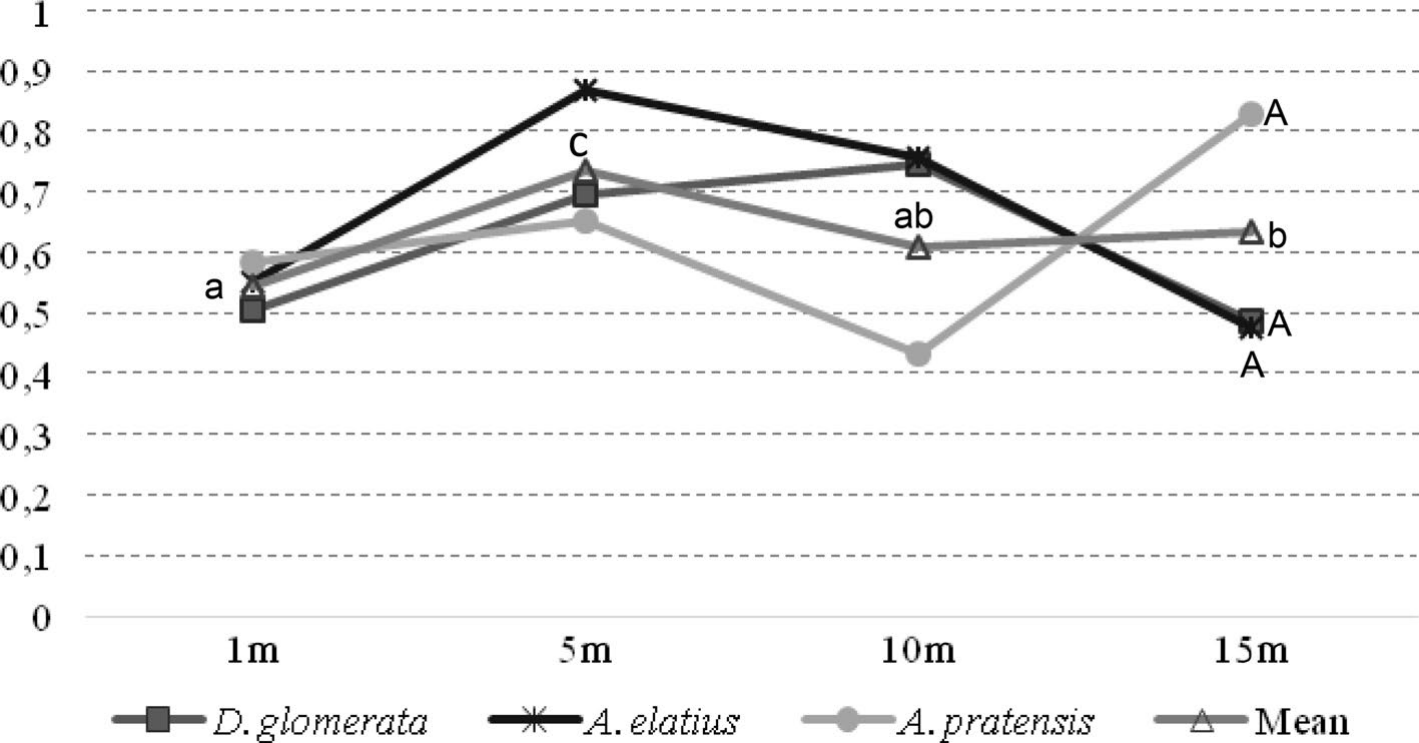
The ratio shoot/root (*M*_*S*_/*M*_*R*_) in Pb content

**Fig. 4:**
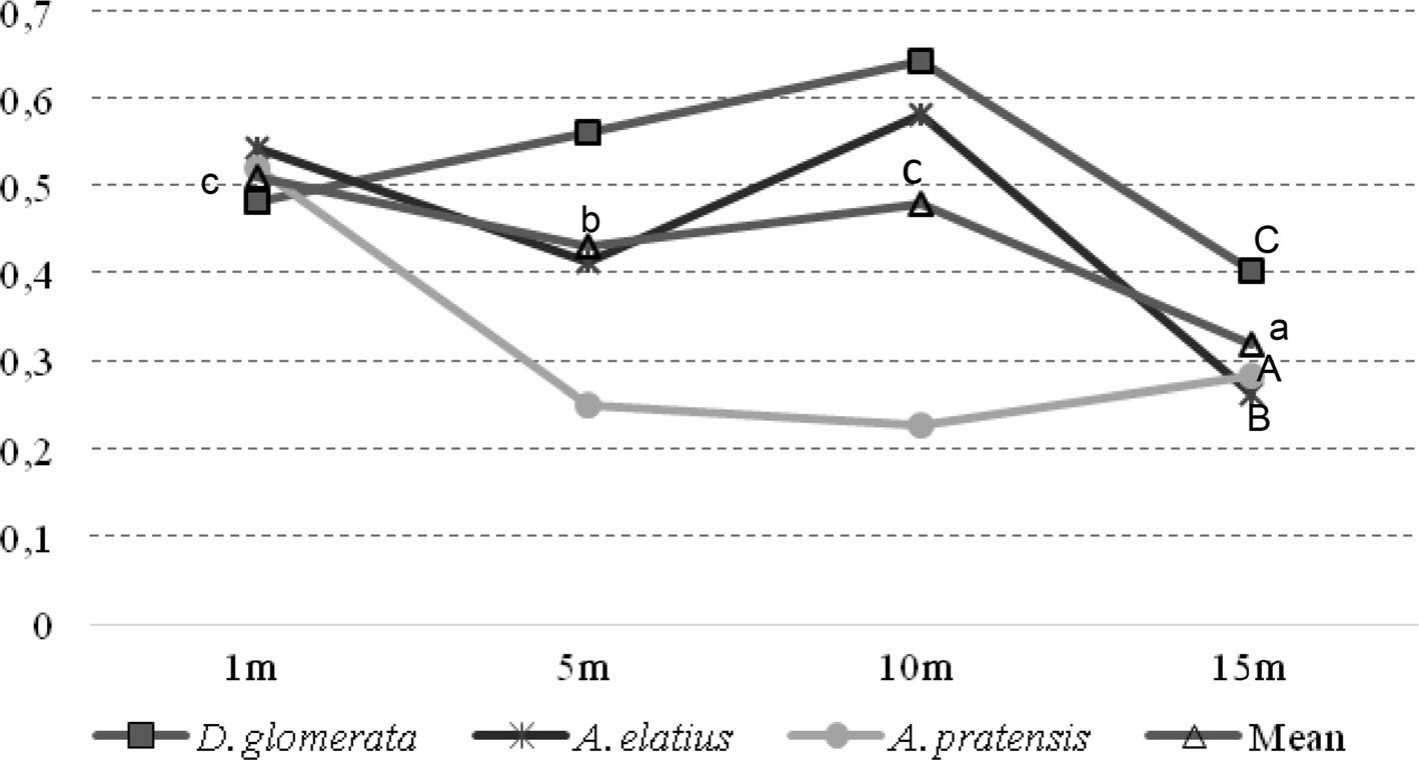
The ratio shoot/root (*M*_*S*_/*M*_*R*_) in Cd content

The ratios of Pb and of Cd accumulation in aboveground parts to their accumulation in roots (MS/MR) are presented in Figs. 3 and 4. For all grass species, there were no significant differences between the average values of the Pb ratio.

However, there were significant differences in the ratio of Pb content between plant samples collected at different distances. The ratio value for Cd showed different responses of each grass species to the release of heavy metals by traffic. The lowest value of this indicator was reported in *A*. *pratensis* and the highest in *D*. *glomerata*. In the case of Cd, there was a considerable variation of those values depending on the distance from the road but also on overall Cd content in the plant. In the case of Pb ratio, the values were higher than in the case of Cd.

Comparing grass species containing the highest amounts of Pb and Cd (Table 5), it was found that the greatest concentration of Pb were accumulated by *D*. *glomerata* growing 1 and 5 m from the road and by *A*. *pratensis* growing 10 and 15 m from the road. However, Cd was accumulated by *D*. *glomerata* in high quantities at all distances, except 10 m from the road where *A*. *elatius* had the highest concentration of this chemical element.

**Table 5:** Grass species with the highest and lowest accumulation of Pb and Cd

Distance from the road (m)	Maximum		Minimum	
	Pb	Cd	Pb	Cd
1	*Dactylis glomerata*	*Dactylis glomerata*	*Arrhenatherum* *elatius*	*Alopecurus* *pratensis*
5	*Dactylis glomerata*	*Dactylis glomerata*	*Alopecurus* *pratensis*	*Alopecurus* *pratensis*
10	*Alopecurus* *pratensis*	*Arrhenatherum* *elatius*	*Dactylis glomerata*	*Alopecurus* *pratensis*
15	*Alopecurus* *pratensis*	*Dactylis glomerata*	*Arrhenatherum* *elatius*	*Arrhenatherum* *elatius*

The lowest content of both Pb and Cd varied and was dependent on both the type of grass and the distance from the road. There was no clear tendency for the same species to have the lowest Pb content at a few distances from the road. In turn, the smallest amounts of Cd were found primarily in *A*. *pratensis* growing 1 to 10 m from the road, whereas at a distance of 15 m, its smallest content was found in *A*. *elatius*.

The calculated values of plant accumulation coefficients (the ratio of heavy metal concentration in plants to heavy metal concentration in the soil) for Pb and Cd varied (Tables 6 and 7). Those values for Cd were much higher than for Pb. Much higher accumulation coefficient values for Pb and Cd were observed in underground parts of grass species. In the case of Cd, the coefficient was the highest for *D*. *glometara* (2.456), whereas *A*. *pratensis* had the highest Pb coefficient (0.623). The average value for all species was the highest at 15 m from the road for Cd and at 1 m for Pb. The findings of the present experiment indicated that in the case of Pb the average coefficient value for all grass species, and distances from the road were much lower than that provided by Malinowska and Jankowski (2016).

**Table 6:** Accumulation coefficient for Pb and Cd in aboveground parts of selected grass species

Distance from the road (m)	Grass species	Accumulation coefficient	
		Pb	Cd
1	*D. glomerata*	0.405	1.250
	*A. elatius*	0.282	0.825
	*A. pratensis*	0.393	0.503
5	*D. glomerata*	0.350	1.610
	*A. elatius*	0.288	0.613
	*A. pratensis*	0.258	0.562
10	*D. glomerata*	0.279	1.080
	*A. elatius*	0.291	1.570
	*A. pratensis*	0.424	0.686
15	*D. glomerata*	0.233	0.928
	*A. elatius*	0.104	0.454
	*A. pratensis*	0.449	0.914
Means for each distances			
1		0.360	0.859
5		0.297	0.928
10		0.331	1.11
15		0.262	0.765
Means for each species			
*D. glomerata*		0.317	1.217
*A. elatius*		0.241	0.866
*A. pratensis*		0.381	0.666
Means for element			
		0.313	0.916

**Table 7:** Accumulation coefficient for Pb and Cd in underground parts of selected grass species

Distance from the road (m)	Grass species	Accumulation coefficient	
		Pb	Cd
1	*D. glomerata*	0.683	2.587
	*A. elatius*	0.486	2.010
	*A. pratensis*	0.630	2.254
5	*D. glomerata*	0.500	2.369
	*A. elatius*	0.453	1.282
	*A. pratensis*	0.435	1.550
10	*D. glomerata*	0.470	2.281
	*A. elatius*	0.500	2.629
	*A. pratensis*	0.649	2.252
15	*D. glomerata*	0.435	2.586
	*A. elatius*	0.327	1.921
	*A. pratensis*	0.687	3.079
Means for each distance			
1		0.630	2.284
5		0.463	1.734
10		0.540	2.387
15		0.483	2.529
Means for each species			
*D. glomerata*		0.522	2.456
*A. elatius*		0.442	1.961
*A. pratensis*		0.623	2.284
Means for element			
		0.529	2.234

**Fig. 5:**
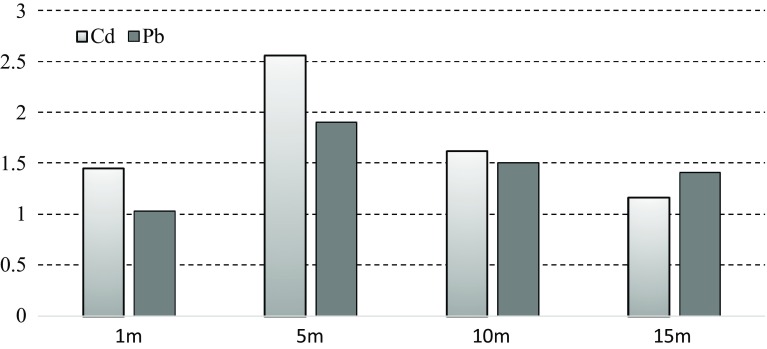
Soil pollution indices for Pb and Cd in the vicinity of the E30 expressway

The pollution index (*P*_*i*_) quite is often used to assess soil contamination in areas close to expressways (Christoforidis and Stamatis 2009). The value of the index for Cd and Pb was above 1 in all measuring points, which meant medium-level pollution (Fig. 5). In the soil, the largest value of the pollution index (*P*_*i*_) for both metals was 5 m from the road.

Simple correlation coefficient values showed no significant interaction between Pb content in the plants and in the soil (Table 8). However, there was a significant positive correlation between Cd content in the soil and in the aboveground and underground parts of *D*. *glomerata*.

**Table 8:** Simple correlation coefficient between the overall content of Pb and Cd in the soil and in the above-ground and underground parts of selected grass species

Species	Pb	Cd
Aboveground parts		
*Dactylis glomerate*	0.735	0.990*
*Arrhenatherum elatius*	0.676	0.371
*Alopecurus pratensis*	0.303	0.760
Underground parts		
*Dactylis glomerate*	0.735	0.990*
*Arrhenatherum elatius*	0.822	0.371
*Alopecurus pratensis*	0.111	0.770

*p* ≤ 0.05

*Indicates significant difference

## Discussion

This study presents the results of Pb and Cd content in the soil and grasses sampled at different distances from the expressway. In the soil, the average content of Pb and Cd was 10.37 and 0.221 mg kg^−1^, respectively. According to Martin et al. (2017) and Kabata-Pendias and Pendias (1999), such amounts were not high. The natural concentration of Cd in soils of the world ranges from 0.20 to 1.05 mg kg^−1^, usually not exceeding 0.5 mg.kg^−1^ Cd (Alloway 2013), whereas in Poland it ranges from 0.03 to 0.22 mg kg^−1^ Cd (Kabata-Pendias 2004).

In the present study in the aboveground parts of grasses, the greatest amount of Pb (3.843 mg kg^−1^DM) was absorbed by *Aloperucus pratensis* and the smallest (3.246 mg kg^−1^DM) by *Dactylis glomerata*. Other studies (Viard et al. 2004) have found that Pb concentration in grass species (*Festuca arundinacea*, *Phalaris* species, *D*. *glomerata* L.) growing close to main roads ranged from 1.0 to 2.0 mg kg^−1^DM, whereas in samples collected 5 and 20 m away from the roadway, it was in the range of 0.8-2.2 and 0.5-0.7 mg kg^−1^DM, respectively. Some studies (Filipek-Mazur et al. 2007) performed in different parts of Poland have found that the average concentration of Pb in grass species was 2.5 mg kg^−1^DM, ranging from 0.6 to 15 mg kg^−1^DM. In other countries the average Pb concentration in grass species ranged from 0.4 (Finland) to 4.6 mg kg^−1^DM (Kazakhstan); as said above, in Poland the highest Pb concentration amounted to 15 mg kg^−1^DM (Filipek-Mazur et al. 2007). In the present study, Cd content in aboveground parts of grass ranged from 0.069 mg kg^−1^DM in *A*. *elatius* sampled at a distance of 15 m from the road to 0.537 mg kg^−1^DM in *D*. *glomerata* collected 5 m from the road. In some studies (Viard et al. 2004), Cd concentration in green parts of grass collected 5 and 20 m from the road was similar and ranged from 0.03 to 0.07 mg kg^−1^DM. In other countries, Cd concentration in grass ranged from 1.0 to 1.6 mg kg^−1^DM (Belgium) and from 0.3 to 2.9 mg kg^−1^DM (Hungary). In Poland, the concentration of this chemical element varied between different species of grass in the range from 0.1 to 2.6 mg kg^−1^DM, whereas for *D*. *glomerata* L., these values ranged from 0.05 to 0.80 mg kg^−1^DM (Jankowski et al. 2015). According to Kabata-Pendias (2004), the maximum limits of the content of different heavy metals in forage are as follows: < 100 mg Zn < 30 mg Cu < 20 mg Cr < 50 mg Ni < 10 mg Pb < 0.5 mg Cd kg^−1^DM. In the present study, the content of Pb and Cd in the biomass of the grass species was within the above limits.

The ratio value for Pb and Cd showed different responses of each grass species to the release of heavy metals by traffic. The lowest value of Cd was reported in *A*. *pratensis* and the highest in *D*. *glomerata*. Underground parts of the grass species accumulated greater amounts of Pb and Cd than aboveground parts, which was indicated by accumulation coefficient values of 0.529/0.313 and 2.234/0.916, respectively. In another study (Dai et al. 2004), three species of grass were exposed to extremely high levels of Pb and Cd, with the ratio showing slightly different values from those of the present experiment. In the case of Pb, it ranged from 0.01 to 0.02 and for Cd it was from 0.12 to 0.50. The calculated values of accumulation coefficient for Cd were much higher than for Pb. Additionally, Kloke et al. (1984) reported that of all heavy metals Cd had the greatest accumulation coefficient (1–10) and Pb the smallest (0.01–0.1). According to Kloke et al. (1984) and Kabata-Pendias and Pendias (1999), a coefficient value higher than 1 is a proof of intense accumulation of a metal by plants. Only in the case of the aboveground parts of *D*. *glomerata* was the coefficient higher than 1 (1.217). The present study indicates that considerable amounts of heavy metals that were released by vehicles on the expressway contaminated the soil.

## Conclusions

Grass species tested in the experiment, namely *D*. *glomerata* L., *A*. *pratensis* L., and *A*. *elatius* L., had varied abilities to accumulate heavy metals. The largest amounts of Pb were absorbed by *A*. *pratensis* L. (3.843 mg kg^−1^ DM) and the lowest by *A*. *elatius* L. (2.523 mg kg^−1^DM). Of the above-mentioned plants, the highest amount of Cd (0.286 mg kg^−1^DM) was accumulated by *D*. *glomerata* L.

Underground parts of the grass species accumulated greater amounts of Pb and Cd than aboveground parts, which was indicated by the accumulation coefficient values of 0.633 and 0.436, respectively. It indicates that considerable amounts of heavy metals released by vehicles on the expressway contaminated the soil.

The distance from the road clearly affected the content of heavy metals in the grass species. The highest amount of Pb and Cd was found in the grass growing at a distance of 5 m from the edge of the roadway, and this applies both to underground and aboveground parts. The results point to a need to monitor plant contamination by chemical elements released from road traffic, with a view to possible restrictions on the use of such plants as animal feed or medicine.
